# Supplementary material for: Activations of deep convolutional neural networks are aligned with gamma band activity of human visual cortex
Source: Commun Biol. 2018 Aug 8;1:107. doi: 10.1038/s42003-018-0110-y (PMC6123818; doi:10.1038/s42003-018-0110-y)
Supplement: Supplementary file 1 — Supplementary Information [file 42003_2018_110_MOESM1_ESM.pdf]

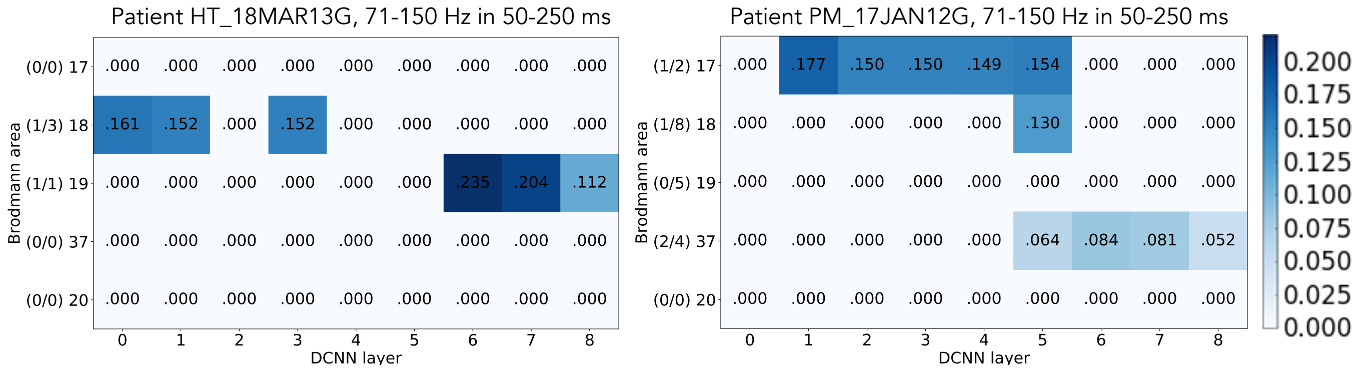

Supplementary Figure 1. Single subject results from two different subjects. The numbers show the sum of correlations normalized by the number of probes in an area. On the left plot we see how a probe in Brodmann area 18 is mapped to the layers 0, 1, and 3 DCNN, while the activity in Brodmann area 19, which is located further along the ventral stream, is mapped to the higher layers of DCNN: 6, 7, 8. Similar trend is seen on the right plot. The numbers on the left of each subplot show the number of significantly correlating probes in each area out of the total number of responsive probes in that area.

	θ	α	β	γ	Γ
$50-250$ ms	1299	709	269	348	504
$150-350$ ms	1689	783	260	515	745
$250-450$ ms	1687	802	304	555	775

Supplementary Table I. Number of positively responsive electrodes in each of the 15 regions of interest in a time-resolved spectrogram.
